# Direct Approach or Detour: A Comparative Model of Inhibition and Neural Ensemble Size in Behavior Selection

**DOI:** 10.3389/fnsys.2021.752219

**Published:** 2021-11-09

**Authors:** Trond A. Tjøstheim, Birger Johansson, Christian Balkenius

**Affiliations:** Department of Philosophy, Lund University Cognitive Science, Lund, Sweden

**Keywords:** detour task, egocentric navigation, allocentric navigation, navigational strategy selection, consciousness, inhibition

## Abstract

Organisms must cope with different risk/reward landscapes in their ecological niche. Hence, species have evolved behavior and cognitive processes to optimally balance approach and avoidance. Navigation through space, including taking detours, appears also to be an essential element of consciousness. Such processes allow organisms to negotiate predation risk and natural geometry that obstruct foraging. One aspect of this is the ability to inhibit a direct approach toward a reward. Using an adaptation of the well-known detour paradigm in comparative psychology, but in a virtual world, we simulate how different neural configurations of inhibitive processes can yield behavior that approximates characteristics of different species. Results from simulations may help elucidate how evolutionary adaptation can shape inhibitive processing in particular and behavioral selection in general. More specifically, results indicate that both the level of inhibition that an organism can exert and the size of neural populations dedicated to inhibition contribute to successful detour navigation. According to our results, both factors help to facilitate detour behavior, but the latter (i.e., larger neural populations) appears to specifically reduce behavioral variation.

## 1. Introduction

Navigation through space, including taking detours, is an essential element of consciousness (Klein and Barron, 2016; Mallatt et al., 2021). Therefore, exploring the basic mechanisms of these behaviors contributes to the study of consciousness, even if the early steps in the evolution of animal navigation were algorithmic-like and lacking in subjective consciousness like in the model presented in this study. When an organism can no longer follow gradients but must use memory and map-like cognitive structures to cope with an environment, that organism comes closer to supporting a representation of space that is not centered on itself. That is, it supports allocentric representations in addition to self-centered, or egocentric representations. The former affords to see the self in relation to the environment, like being *behind* a tree or *to the east* of a river. The latter affords direct movement like going *forward* or turning to the *right*.

**Figure 1:**
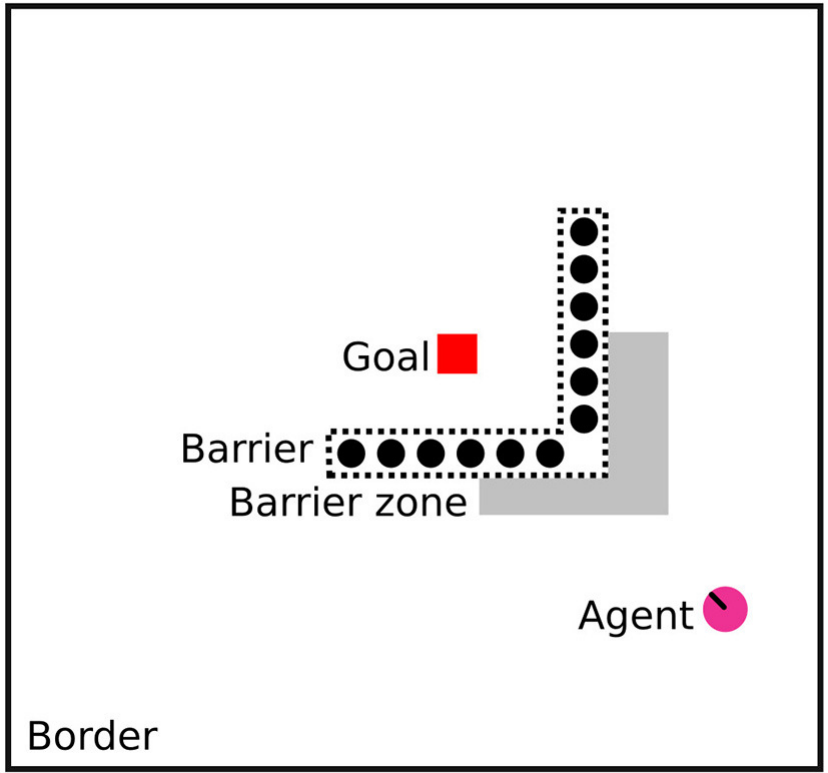
Configuration of the detour task used in experiments, to scale. The barrier is semitransparent with vertical opaque stripes, and the agent is placed facing the apex of the barrier. The diagram shows the goal in red, the semitransparent barrier with transparent parts with dotted lines, the barrier zone in gray, the agent in pink, and the surrounding border.

Natural environments may require a diverse number of behavioral strategies to yield optimal access to resources, while balancing safety and competition concerns. However, this variety can often be condensed into two major types mentioned above; allocentric map-based navigation or egocentric direct approach (Bottini and Doeller, 2020). The extent to which species are biased toward egocentric or allocentric navigation is typically dependent on ecological factors like food availability and the availability of sensory cues (Bruck et al., 2017). Much work has been done to compare species with regards to their ability to control the urge to directly approach salient targets like food, mates, or social groups, and be able to navigate around obstacles *via* detour paths (Kabadayi et al., 2018). In psychology and ethology, this kind of behavior is investigated using detour tasks. The idea of these experimental tasks is that an animal cannot directly approach a target, but must navigate or reach around a barrier first (As shown in Figure 1). In the case of navigation tasks, there is usually defined a *barrier zone* immediately in front of the barrier, and the time the animal spends in this zone can be used to operationalize an experimental measure of its inhibitory control, which is the ability to inhibit a futile direct approach and then take a detour.

Kabadayi et al. (2018) review how detour tasks are used in animal cognition. They enumerate the various configurations, measurements, and animal species that have so far been employed in this context. According to them, the behaviors of a wide variety of families of species have been measured, including apes (*homo* and hominoidae), monkeys (cercopithecoidae and platyrrhini), lemuriforms (strepsirrhini), canids (canidae), equids (equidae), birds (aves), reptiles (reptilia), amphibians (amphibia), fish (pisces), molluscs (mollusca), and spiders (salticidae). Detour tasks have also been used to elucidate the characteristics of several cognitive capacities that include inhibitory control, insight learning, memory, motor and cognitive development, functional generalization, and social learning.

As mentioned, Kabadayi et al. (2018) enumerate several configurations of the detour task. One of these is the V-shaped semitransparent configuration. This has been used to test social learning, problem solving, and inhibitory control in several canids such as dingos, dogs, and wolves, as well as mammals like mice, and goats, and reptilians like tortoises. For mice, the configuration is typically adapted to have a circular border and be filled with water, while the goal is a platform that allows subjects to escape from submersion. This is in contrast with e.g., canids, where the goal is a reward like food or social interaction. Subjects can either be placed inside the V barrier and having to move out of it (outward task), or outside it, having to move in (inward task). Refer to Figure 1 for an example of the inward task which is used in this study. The outward task is usually taken to be the more challenging one as it typically requires subjects to move in the opposite direction to the goal.

Focusing on inhibitory ability and behavioral control in the inward, semitransparent V configuration of the detour task, Marshall-Pescini et al. (2015) investigated how wolves (Canis lupus) and dogs (Canis lupus familiaris) differ in this configuration, seeking to test which species can exhibit better inhibitory control. They found that wolves showed shorter latency to reach the goal, and persevered for less time at the barrier. However, dogs had the upper hand in the so-called cylinder task where subjects are required to get at the reward by gaining access through the opening of a cylinder. It is notable that Bray et al. (2015) found that differences appear to exist between dogs with different levels of excitability, or temperament. Comparing calm and excitable dogs, their findings indicate that calm dogs improved their success rate and apparent inhibitory control with increasing arousal, while excitable dogs performed poorer. Juszczak and Miller (2016) employed the V-shaped detour task placed in shallow water to investigate detour behavior in mice. They measured time in the barrier zone in front of the barrier, for both transparent and semitransparent barriers. In their tests, the performance of the mice appeared to depend both on individual inhibitory skills and experience with the task. That is, they found that performance tended to improve over time, and the mice spent less time in the barrier zone as they gained experience.

The ability to change behavior and strategies for approach as presented above is referred to as behavioral flexibility (e.g., Coppens et al., 2010). As the animal studies explain, behavioral flexibility is thought to involve inhibitory activity to balance the influence both of learned behavior and approach motivation toward salient reward stimuli in the immediate environment. For humans, Uddin (2021) identified large-scale functional brain networks encompassing lateral and orbital frontoparietal, midcingulo-insular and frontostriatal regions that support flexibility across the lifespan.

Spiers and Gilbert (2015) propose a conceptual model in which the lateral prefrontal cortex (PFC) provides a prediction error signal about the change in the path, the frontopolar and superior PFC support the re-formulation of the route plan as a novel subgoal and the hippocampus (HC) simulates the new path. Similarly, the ventromedial (vm) PFC may mediate between the conflicting behavioral responses indicated by HC or caudate systems when active (Doeller et al., 2008). The caudate nucleus is involved in landmark-based, egocentric navigation, while HC is involved in boundary-based, allocentric navigation. According to Piray et al. (2016), the strength of the vmPFC projection to medial striatum including the caudate nucleus, biases toward model-centric choices. Model-centric strategies are typically associated with allocentric navigation (Doeller et al., 2008). These circuits for navigation present contingent behavioral sequences that can be activated. Which of them will be chosen at any given time is dependent on separate machinery, as explained below.

Neural competition is a cornerstone of many theories of brain function, particularly for processes involved in selection and decision making (Amari, 1977; Grossberg, 1978; Erlhagen and Schöner, 2002). Leaky competing integrator models incorporate aspects of both the psychological and neurophysiological models (Usher and McClelland, 2001, 2004; Johnson and Ratcliff, 2014). Relating to this, Smith (2015) shows that the precision of neural populations increases with the number of participating neural units. In the experiments presented in their study, they used units designed to behave according to an idealized Poisson process, having an exponentially decreasing probability of activity after a stimulus. In the context of visual short term memory, they showed in particular that the signal-to-noise ratio (i.e., the precision) increases proportionally to the square root of the neuronal population size. They also showed that normalization of inputs can be achieved by shunting inhibition, which in practice allows fractional scaling of inputs without losing temporal signatures of signals (Prescott and De Koninck, 2003). According to them, their population-size-dependent normalization model allows theoretical models of reaction time and decision accuracy to be reconciled with experimental data.

Earlier we focused on arousal levels in the context of the noradrenergic system (Balkenius et al., 2018), and found that neural gain in the form of noradrenergic activation may contribute to switching between explorative and exploitative behavioral strategies by e.g., varying the amount of noise present in the selection process. In this study, we concentrate on the effect of varying the size of neural populations, and how that affects precision and integration of sensory information. Additionally, we explore how inhibitive efficacy and precision individually and together can contribute to behavioral strategy selection. Finally, we compare our results with data from experiments on animal species, specifically mice, dogs, and wolves.

## 2. Method

In this section, we explain the rationale behind the model, its properties, and how in particular it is implemented.

### 2.1. Properties of the Model

**Figure 2:**
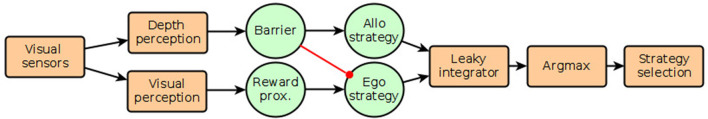
Diagram showing model of strategy selection. Green circular objects represent neural populations that receive signals from perceptual modules. Neural populations are simulated with different numbers of neural units, as described in the text. The red connection indicates inhibition, the level of which is varied between 0 and 1 in simulations. The “Barrier” population is excited by barriers or obstacles immediately in front of the agent, while the “Reward proximity” population is excited by the width of red-colored objects in the visual field.

To allow selection between the two strategies of egocentric direct approach and allocentric detour, we appropriated a hypothesized network proposed by Barker and Baier (2015). This was originally suggested as a model of approach and avoidance behavior in fish. But given appropriate input signals, it can be used as a winner-takes-all network to select between strategies for approach. In particular, we added one-way inhibition between barrier-collision signals to the neural units representing egocentric strategy. This modified network architecture (as shown in Figure 2 for a diagram) is informed by work on the spatial pathway from the parietal cortex to vmPFC (Kravitz et al., 2011) that includes boundary sensitive cells in the subiculum (Epstein et al., 2017), and projections from vmPFC to the subthalamic nucleus that can inhibit impulsive behavior (Eagle and Baunez, 2010). The variation of population size and inhibitive strength is likewise informed by Smith (2015) and Piray et al. (2016), respectively.

The spiking model used for the neural elements is as defined by Izhikevich (2003):


(1) $$\begin{array}{r} \frac{dv}{dt}=0.04v^{2}+5v+140-u+I \end{array}$$

(2) $$\begin{array}{r} \frac{du}{dt}=a\left(bv-u\right) \end{array}$$

(3) $$\;v\text{ =}\left\{ \begin{array}{l} c,\text{ if }v=30\text{mV}\\ v,\text{ otherwise} \end{array}\right.$$

(4) $$u=\left\{ \begin{array}{l} u+d,\text{ if }v=30\text{mV}\\ u,\text{ otherwise} \end{array}\right.$$

In this study, Equations (1, 2) define pre-spike behavior, while Equations (3, 4) define the reset behavior after a spike. In Equation (1), *I* is for direct input current; *v* is the voltage potential of the unit, and *u* is a negative feedback variable to *v* accounting for positive ionic currents. Refer to Table 1 for parameter values for *a*, *b*, *c*, and *d*; these values are in accordance with “regular firing” units as defined in Izhikevich (2003).

**Table 1:** Numerical values used for simulation.

**Parameter**	**Value**
*a*	0.02
*b*	0.2
*c*	–65.0 + 15 γ^2^
*d*	8–6 γ^2^
ω	0.024
ϵ	0.1
λ	0.9
τ	1

*Parameters, a, b, c, and d are used for the simulation of spiking units. γ is a noise term between 0 and 1 used to slightly randomize spiking units, as described in Izhikevich (2003). ω, ϵ, λ, and τ are used for the leaky integrator*.

The formula for the leaky integrator is given by:


(5) $$\begin{array}{r} y_{t+1}=e\left(x-\left(1-l\right)y_{t}\right) \end{array}$$

where *y* is the value of the integrator, *e* is the growth or decay factor (as shown below), *x* is the input, and *l* is the leakage factor that affects accumulation. These are defined as follows:


(6) $$e\text{ =}\left\{ \begin{array}{l} \omega ,\text{ if }x<\tau \\ \epsilon ,\text{ otherwise} \end{array}\right.$$

(7) $$l=\left\{ \begin{array}{l} 0,\text{ if }x<\tau \\ \lambda ,\text{ otherwise} \end{array}\right.$$

Equations (6, 7) define the behavior of the integrator when the input is less than the decay threshold τ. At this point, the integrator begins leaking, or decaying in value, and the value of *e* changes from ϵ to ω. Refer to Table 1 for numerical values for these parameters.

### 2.2. Implementation

The neural simulation model was implemented using the Processing framework v.3.5.3 (Reas and Fry, 2007) with the pOSC library v.0.9.9, while the agent and environment were implemented in the Unity game engine v.3.5. Refer to Figure 1 for task configuration in Unity. The neural simulation and the agent world were connected using the Open Sound Control (OSC) protocol (Wright and Freed, 1997). In this way, the agent sends out sensory signals while the neural simulation processes these signals, and computes a motor response that is transmitted back and executed by the agent. This back-and-forth communication happens continuously and asynchronously. The set of signals is described in Table 2.

**Table 2:** List of OSC messages used to communicate state of agent in simulated environment.

**Signal**	**Description**
/camera_r	Red channel from camera
/depth/camera	Depth rendering from camera
/borders	The position and size of the border walls
/obstacles	The position and size of the obstacles
/goals	The position and size of the goal
/agents	The position and size of the agent
/config	An int denoting the current task configuration
/camera/rotation	The relative camera rotation since last step
/camera/absrotation	The absolute camera rotation
/ready	A signal telling the neural simulation that agent is in the initial position and can receive motor commands
/barrierareas	The position and size of the barrier areas

The simulation supports two-approach strategies; egocentric direct approach and allocentric approach using a map. The former is implemented by slicing a vector of pixels from the color channels of the cameras, then using pixels from the green and blue channels to remove anything but the purely red pixels in the vector from the red channel. The red pixels are counted, and their center point is calculated. Together, this yields a weighted homing signal that can be used for a direct approach such that the sensor information and the motor signals together form a feedback control circuit.

The allocentric map navigation is based on the classical wavefront algorithm (WFA) (Dijkstra, 1959). To facilitate the building of wavefront maps, the agent world sends bounding boxes of all necessary borders, obstacles, and goals, as well as the position of the agent itself. These bounding boxes are used to render a matrix of binary values, making up a map of the environment that can be used by the WFA. The WFA then calculates a gradient from the goal to the agent at every simulation step (to tell if it is getting closer), which gives the agent a direction to move in. This enables the agent to take detours around the obstacles.

As a source of bias for the allocentric strategy, we sliced a vector from the middle of the depth texture from the camera, and transformed it into a two-dimensional matrix. The four topmost rows of this matrix then represent obstacles at various distances from the agent. The rows were weighted and summed up, and the resulting sum was used as a direct input to the spiking population named “Barrier” in Figure 2. The spatial pixel density, thus, forms a kind of receptive field similar to those associated with boundary and obstacle cells in medial temporal areas (Epstein et al., 2017; Poulter et al., 2018). Similarly, the aforementioned sum of red pixels taken from the color camera was used as direct input to the parallel spiking population named “Reward proximity” in Figure 2. These populations were connected to populations representing either the allocentric strategy or the direct approach strategy, with the output of the obstacle bias also connected to the direct approach unit *via* an adjustable inhibitory weight. Again, refer to Figure 2 for a diagram of the network. The output of the two strategy units was connected to leaky integrator units to be able to transform the spiking trains to scalars suitable for identifying the index of the channel with the largest value (argmax selection). This index was then used to select the winning motor commands for transmission to the agent motor system.

During experiments, the level of inhibitory weight was controlled and set to progressively be from zero to one (refer to Table 3). The agent was given a starting point in view of the target (refer to Figure 1), then left to find its way. The maximum number of steps was set to 1,200, and the simulation was run at 10 Hz, giving a maximum time of 120 s. This makes it possible to compare times in seconds with animal experiments (120 s was also the maximum time limit used for dogs and wolves in Bray et al., 2015). A successful approach to the target was defined as the agent coming within a set radius (5 world units) of the center of the target. After reaching the goal, or the time limit being exceeded, the simulation was reset, parameters for the spiking units were slightly randomized (refer to Table 1), and the agent returned to its initial position. Fifteen trials like this were carried out for each inhibitory weight and neuron population size pair. The information gathered from each trial is given again in Table 3, and the data was then used to produce statistics.

**Table 3:** Summary statistics for simulations with varying population size and inhibition level, listing summary statistics including mean with SD, median with interquartile range (IQR), as well as minimum and maximum values.

**Population size**	**Inhibition**	**Mean**	**SD**	**Median**	**IQR**	**Min**	**Max**
1	0.00	26.39	31.03	12.40	8.42	7.40	110.90
1	0.10	11.44	7.82	9.25	5.83	5.50	35.60
1	0.20	15.59	19.95	5.95	4.20	4.40	70.90
1	0.40	10.36	8.34	6.85	5.40	4.30	33.70
1	0.60	5.82	1.49	5.70	1.50	2.20	8.20
1	0.80	5.77	3.88	4.85	0.65	3.20	17.60
1	1.00	5.28	2.19	4.50	1.80	2.10	11.30
2	0.00	15.16	17.91	7.35	4.95	5.80	71.70
2	0.10	6.65	3.21	6.00	0.70	4.80	17.70
2	0.20	11.04	20.32	5.30	0.88	4.60	81.50
2	0.40	7.08	7.46	4.85	1.32	4.10	32.70
2	0.60	10.10	11.62	5.80	3.88	3.70	47.40
2	0.80	6.39	3.64	5.10	1.95	3.70	17.80
2	1.00	5.00	0.99	4.90	1.40	3.60	6.90
5	0.00	13.67	16.34	7.10	2.20	2.40	54.70
5	0.10	5.32	1.02	5.70	1.67	3.30	6.60
5	0.20	5.06	1.83	5.05	0.75	2.00	9.60
5	0.40	4.43	1.46	4.40	0.80	3.00	8.70
5	0.60	9.07	15.84	4.20	1.50	2.10	61.40
5	0.80	7.06	7.94	4.65	1.85	2.10	32.40
5	1.00	5.12	1.76	5.30	1.63	2.00	8.20
10	0.00	9.94	7.17	7.20	3.50	5.30	32.30
10	0.10	5.63	0.64	5.70	1.02	4.70	6.50
10	0.20	5.36	1.32	5.05	0.88	4.00	9.50
10	0.40	5.19	1.11	4.90	0.45	4.00	7.90
10	0.60	4.58	1.32	4.45	1.15	3.00	8.60
10	0.80	4.28	1.12	4.25	1.20	2.80	7.30
10	1.00	3.96	1.12	4.25	1.22	1.20	5.30

The statistics was done using Jupyter notebook software (Kluyver et al., 2016), the python programming language (Van Rossum and Drake, 1995), and the Pandas (McKinney, 2010), Seaborn (Waskom, 2021), numpy (Harris et al., 2020), and scipy (Virtanen et al., 2020) libraries.

To calculate the mean and SD of time in the barrier for the animals in **Figure 4**, we used published data from Marshall-Pescini et al. (2015) for dogs and wolves, and Juszczak and Miller (2016) for mice. Our model does not support learning, hence we calculated statistics only for the subset of data that was recorded at the first trial to minimize the effects of learning and experience. Where different barrier configurations were used, we chose only data from the inward-V configuration.

## 3. Results

In this section, we show results suggesting that increasing the population size of spiking neurons in the neural network generally reduces behavioral variability of the agent, while increasing the weight of inhibition tends to reduce waiting time in the barrier zone. Both of these factors work together to consistently favor the allocentric navigation strategy upon detection of a barrier.

**Figure 3:**
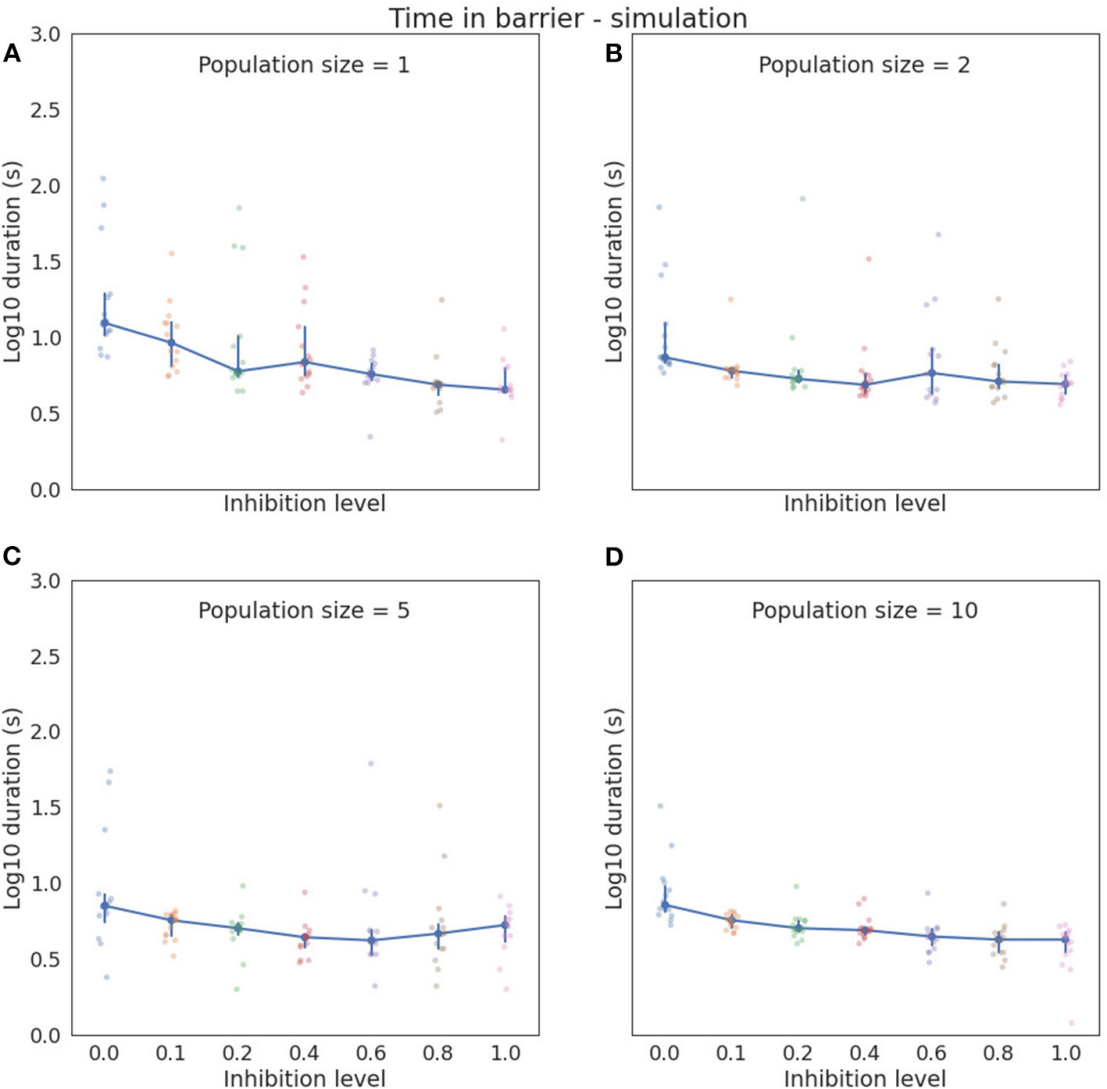
The plot of log10 median time with 95% confidence interval in barrier zone for different simulation configurations. Actual times are indicated by the pale blue and pink dots. **(A)** Simulated neuronal populations each consist of a single neuron. Zero inhibition level yields the highest variance and highest median time in the barrier zone, an while inhibition level of 1 gives the lowest median barrier time. **(B)** Neuronal populations consist of two neurons, **(C)** shows with five neurons, and **(D)** shows with 10 neurons per population. Barrier times and variation generally trend downwards with an increasing number of neurons. Note that median is used instead of mean in these graphs to better accommodate the asymmetric density of the recorded data.

Figure 3 shows barrier wait times for the simulated agents, grouped by inhibition level and the size of the involved neuronal populations. The general trend displayed by this figure is that time in the barrier reduces as the size of the neuronal population grows. Similarly, the variance as indicated by SD reduces. Within a group of the same population sizes, there is an analogous trend of barrier time reduction as inhibition level increases, going from a median of 12.4 (mean = 26.39, SD = 31.03) at zero inhibition and a single neuron per population, to a median of 4.5 (mean = 5.28, SD = 2.19) at inhibition level of one. At the other end of the scale, with 10 neurons per population, the median at zero inhibition is 7.2 (mean = 9.94, SD = 7.17), and 4.25 (mean = 3.96. SD = 1.12) at inhibition of one. It is also noticeable that between the extreme points, both barrier time and variation jump around somewhat for all population sizes except the maximum 10. In this study, the reduction in barrier time is monotonic (as shown in Table 3).

**Figure 4:**
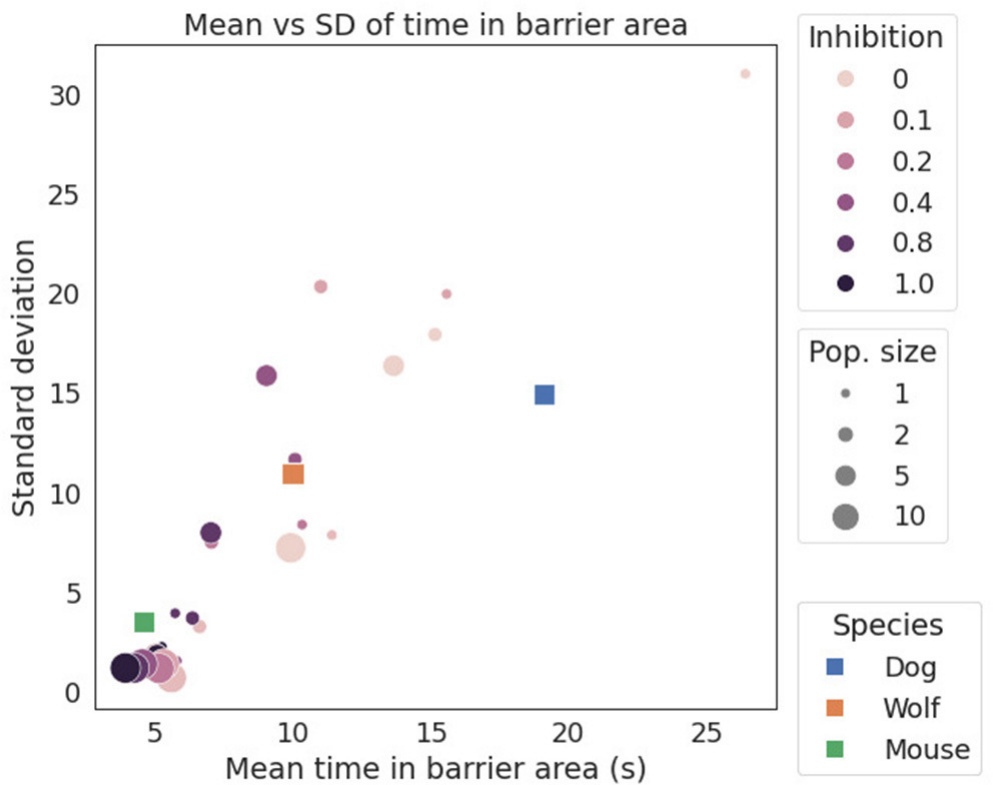
Plot of mean vs. SD for time in barrier zone for different species and simulations. Both the simulation and the animal data appear to be approximately linear. For the three animals, dogs are at the top end of barrier time and variability, and mice at the other extreme. Reducing inhibition yields longer mean time in barrier, while reducing population size increases variability in the form of higher SD. Due to the somewhat stochastic nature of spiking networks, the simulation data naturally displays the noisiness of Figure 3.

Figure 4 shows a scatter plot of mean barrier time vs. SD (i.e., variability). Both animal and simulation data are shown, allowing the animal data to be related to the simulations. Qualitatively, mice spend the least time in the barrier zone and have the least variance, followed by wolves, and with dogs having both the longest time in the barrier, as well as the most variance. Dogs also differ most from the simulated data, spending longer time in the barrier.

## 4. Discussion

In this final section, we first look at possible explanations for the somewhat surprising position of mouse data on our comparative plot and identify stress as one plausible factor. After that, we turn to the role of inhibition in behavior selection, how the ability to make use of allocentric navigation strategies is an elemental part of consciousness, and how inhibition could be of different use to predator and prey species. We then move to some indications that neural population numbers might not automatically predict inhibitive capabilities and discuss how our results might inform findings from animal experiments.

Comparison of behavior between species requires careful controls to take into account differences in anatomy, body structure, and sensory adaptations. Larger bodies tend to require larger brains to control them, and hence direct comparisons of neural numbers are less useful than neural numbers relative to body volume or weight. Another difference between species that can confound comparisons is their dependence on chemical sensation or olfaction. Species for which olfaction is less important are termed *microsmatic*, while those that depend to a large degree on olfaction are termed *macrosmatic* (Santacà et al., 2019a,b). Mice, dogs, and wolves are, hence macrosmatic, while e.g., guppies are considered microsmatic (Santacà et al., 2019a).

One of the interesting inferences one might draw from our results is that mice appear to have more inhibitive powers and larger neuronal populations than do dogs and wolves. One could infer this because mice spend less time at the barrier and more time detouring, so in Figure 4 they group with the high-inhibition and large-population points. This inference, however, is unlikely to be the actual case. Instead, the reason why mice move out of the barrier zone quickly rather than staying like dogs and wolves could be due to the different experimental designs. Mice are averse to being immersed in water, which is a stressor, and they seek the relief of the above-water platform. This means that the mice engage in escape behavior, or avoidance from an aversive stimulus instead of an approach to a rewarding one, as do dogs and wolves. Furthermore, mice are typically aversive to moving into open spaces, which likely also contributes to them spending less time in the barrier zone (e.g., Bailey and Crawley, 2009). According to Schwabe et al. (2010), mice that were subjected to stress preferred an egocentric strategy more often than an allocentric one. Hence, it would be expected that once a goal is detected, they would engage in a direct approach to that goal and, thus, be likely to persevere at the barrier. But the submerged mice in the detour experiments used the allocentric strategy instead. This demands some further explanation: approach and avoidance activate different behavioral pathways in the brain (Namboodiri et al., 2016), where the avoidance pathways are typically less focused on one particular goal-site and instead result in a kind of “anywhere but here” escape behavior (Gray, 1982; Graeff, 1994). In such panic behavior, animals are even prone to crashing into obstacles in an effort to get away. Gray (1982) argues that the mammalian defense system is hierarchical, with the undirected escape system as the most basic one, and which is active at the most acute level of stress. At lower arousal levels with no stress or panic, the behavioral hierarchy allows goal-directed escape. Some support for this hypothesis might come from Juszczak and Stryjek (2019). They found that administering scopolamine to mice tended to increase perseverance behavior and time in the barrier zone. Given that scopolamine inhibits cholinergic activity by antagonistically binding to muscarinic receptors (Birdsall et al., 1978), and that the cholinergic system contributes to the level of arousal, e.g., in fight or flight behavior (Skinner et al., 2004), one interpretation is that the lowered arousal level induced by scopolamine reduces escape motivation enough that the water-stress configuration used for mice becomes more similar to the approach to reward configuration used for other species; i.e., allowing more decision time at the barrier and more time variance in making the decision to detour. Together these factors might explain the surprising position of mice in Figure 4.

Figure 4 shows an approximately linear relationship between mean barrier delay and its variance: more neurons correlate with more inhibition and less delay in successfully choosing to detour. This is in agreement with findings from the animal cognition literature that brain size and neuronal density tend to accompany success rate in tasks that require inhibition (Herculano-Houzel, 2017). Hence, biological neural population numbers can be compared at least relatively to simulated population sizes. This yields the prediction that unstressed mice should display more behavioral variability than dogs in an approach oriented version of the semitransparent V-shaped detour task (i.e., mice in a food-seeking version on dry ground).

Escape behaviors can be automatic, or stimulus-response processes in animals. Such processes are generally believed to be less reliant on consciousness than those necessary for making detours. Consciousness seems to depend on back-and-forth (recurrent) communication between neurons and on the resultant rhythmic synchronization and resonance (e.g., Engel and Singer, 2001; Meador et al., 2002; Engel and Fries, 2016). However, in our model, there are no recurrent connections, and neural populations are not synchronized with rhythmic inhibition. Additionally, as described above, the simulated populations have randomized parameters to explicitly increase activation variance. Hence, there is no direct correlation between neural population activity, and populations are not synchronized. Therefore, the model indicates that synchronizing populations is not necessary to achieve useful signal integration for behavioral strategy selection in navigation.

Behavioral selection without subjective consciousness also appears to be possible through subcortical pathways to the amygdala. These pathways are held to be evolutionarily older than cortical pathways and are found in both fish and reptiles, as well as mammals (McHaffie et al., 2005). For vision, one such pathway projects from the retina, *via* the brainstem superior colliculus and the thalamic pulvinar nucleus, to the amygdala. This pathway is generally assumed to be responsible for phenomena like blindsight, where people with cortical blindness can still guess the position of objects in their near environment. In particular, signals indicating dangerous stimuli, like the presence of snakes and spiders (and angry faces), are mediated *via* this pathway to the amygdala, which can then engage defensive behaviors. Furthermore, it appears that even routine, non-escape behavior like touching the position of a light signal may be supported by subcortical pathways, without requiring conscious perception. This is evidenced by studies on monkeys (Cowey and Stoerig, 1997).

How could we go from a simple, nonconscious allocentric navigation strategy (Figure 2) to one that uses consciousness? Merker (2007) argues that consciousness functionally can be understood *via* a “tripartite” division into (i) target selection (ii) action selection, and (iii) motivational ranking. Although these functions may operate on their own, they typically interact such that motivational ranking can influence target selection, which again can influence the selection of actions. Merker (2007) further argues that these functions need to operate in real time, and that they are integrated *via* a form of simulation. It is this simulation process that effectively constitutes conscious experience. Both target and action selection processes are related to spatial cognition and allow an animal to cope with spatially distributed resources, e.g., that shelter, food, and mates are not all found in the same place. As mentioned above, allocentric maps particularly support navigation to targets that are not directly approachable, or even in the direct vicinity. Hence, a system that allows an animal to be conscious of resource-place associations that are spread out potentially provides evolutionary benefits. Klein and Barron (2016) argue that insect brains may be capable of subjective consciousness since in the proposal of Merker (2007), this is mediated by evolutionary old, subcortical structures like the midbrain and the basal ganglia, and insects have structures that are functionally analogous to these. Similarly, the apparent lack of sufficient spatial perception or sensing in plants is used as a an argument by Mallatt et al. (2021) against plants having consciousness.

Carnivorous predator species and herbivorous prey species have adapted different usage for behavioral inhibition. Whereas, predators could benefit from inhibiting direct approach to prey to avoid detection (Hasson, 1991; Radford et al., 2020), a prey species may use inhibition to stop an approach to potential danger, as well as to “play dead” to reduce attack motivation in a predator (e.g., Gallup et al., 1971). In the case of predators, the perception of an eye pattern in the prey can indicate that the prey is turned in the direction of the predator; this can induce behavioral freeze and change the motivation from a direct approach to detour behavior. This would correspond to the perception of a barrier in our model, and the consequent switch to an allocentric navigation strategy. Similarly, the eyes of predators tend to be front-facing, which is useful for estimating distance (Detwiler, 1955). Prey species, on the other hand, often have side facing eyes since it facilitates surveying larger surrounding areas and hence the detection of potential predators. Although predator and prey species may use inhibition differently to adaptively control behavior, what exactly mediates inhibitory capability in different species is still not completely understood. We turn to this issue next. We have argued above that larger populations of neurons can confer increased precision, but that inhibitive efficacy is not fully dependent on population size. Kabadayi et al. (2017) explored the hypothesis that neuronal population size in the avian pallium might predict success rates on the cylinder task. Given that ravens are very adept at this task, and ravens have a densely populated pallium, they sought to investigate whether other birds with similarly high neural densities perform equally well. Parrots are birds that, like ravens, have comparatively dense palliums. Using parrots as subjects, they did not find evidence for a positive relationship between population size and success on the cylinder task. The parrots performed much poorer than did ravens. The authors interpret these results in two ways. Either that inhibition might not be correlated with pallial neuron count, or that the cylinder task does not measure motor inhibition. Our results lend support to the former of these interpretations (neuron number does not matter in this study) but with a slight twist, namely that there may be differences in inhibitive populations that are independent of total population size but that affect inhibitive efficacy.

Moving from birds to arthropods, Long (2021) compared brain sizes of different spider species and classified the spiders into four groups, where the first group had the smallest brain and the fourth group the largest. Interestingly, a species belonging to the first group, the spitting spider *Scytodes pallidus*, is hunted by a species of the fourth group, the jumping spider *Portia labiata*. Notably, *P. labiata* sometimes changes its hunting strategy depending on whether its prey is a male or female, and whether the female is carrying eggs (Jackson et al., 2002). An egg-carrying female is apparently less dangerous since it must drop its egg to spit. In this case, *P. labiata* makes use of faster, direct-approach strategies. But when hunting a female without eggs, *P. labiata* instead takes longer detours, to attack from behind. This more complex behavior might only be possible due to the larger and more complex brain of *Portia*.

In summary, we have presented a model of navigational strategy selection that shows how a direct approach vs. detour might be influenced by the interplay of both neuronal population size and inhibitive efficacy. The former appears to confer precision that improves signal integration, while the latter facilitates the suppression of direct approach strategies and the usage of allocentric navigation around obstacles. Together both processes contribute to behavioral flexibility in navigating complex environments. Comparing the results presented in this study with data from animal experiences may elucidate differences in inhibitive capabilities in various species.

The work presented in this study opens up several new avenues of exploration and complements earlier simulation work we have presented on awareness (Balkenius et al., 2018) and memory (Balkenius et al., 2020). Combining the present study with the former might further elucidate processes of arousal and how they might affect navigation and behavioral selection in the context of making detours. The latter work on episodic memory and decision making offer exciting opportunities for exploring path-learning and how an agent might react when such paths are changed. In the animal cognition literature, the mechanism by which animals are able to take advantage of shortcuts is an example of this that is of particular interest.

## Data Availability Statement

The code for the simulations are publicly available. This data can be found here: https://github.com/trondarild/Tjostheim_et_al_direct_approach_inhibition.

## Author Contributions

TT conducted simulations and wrote the manuscript in collaboration with and under the supervision of BJ and CB. All authors contributed to the article and approved the submitted version.

## Funding

This study was partially supported by the Wallenberg AI, Autonomous Systems and Software Program–Humanities and Society (WASP-HS) and funded by the Marianne and Marcus Wallenberg Foundation and the Marcus and Amalia Wallenberg Foundation.

## Conflict of Interest

The authors declare that the research was conducted in the absence of any commercial or financial relationships that could be construed as a potential conflict of interest.

## Publisher's Note

All claims expressed in this article are solely those of the authors and do not necessarily represent those of their affiliated organizations, or those of the publisher, the editors and the reviewers. Any product that may be evaluated in this article, or claim that may be made by its manufacturer, is not guaranteed or endorsed by the publisher.
